# Association between the *PPP1R3B* polymorphisms and serum lipid traits, the risk of coronary artery disease and ischemic stroke in a southern Chinese Han population

**DOI:** 10.1186/s12986-018-0266-y

**Published:** 2018-04-12

**Authors:** Wei-Jun Li, Rui-Xing Yin, Jian-Hua Huang, Yuan Bin, Wu-Xian Chen, Xiao-Li Cao

**Affiliations:** 1 Department of Cardiology, Institute of Cardiovascular Diseases, the First Affiliated Hospital, Guangxi Medical University, Nanning, 530021 Guangxi People’s Republic of China; 2 Department of Neurology, the First Affiliated Hospital, Guangxi Medical University, Nanning, 530021 Guangxi People’s Republic of China

**Keywords:** Protein phosphatase 1 regulatory subunit 3B gene, Single nucleotide polymorphisms, Lipid, Coronary artery disease, Ischemic stroke, Haplotypes, Interaction

## Abstract

**Background:**

Little is known about the association of the protein phosphatase 1 regulatory subunit 3B gene (*PPP1R3B*) single nucleotide polymorphisms (SNPs) and serum lipid levels, the risk of coronary artery disease (CAD) and ischemic stroke (IS) in the Chinese populations. This study detected such association in a Southern Chinese Han population.

**Methods:**

Genotypes of 4 novel *PPP1R3B* SNPs (rs12785, rs330910, rs330915 and rs9949) in 1704 Han Chinese (CAD, 556; IS, 531 and control, 617) were determined by the Snapshot technology.

**Results:**

The rs12785A and rs9949A allele frequency was higher in both CAD/IS patients than in controls. The rs330910T and rs330915T allele frequency was also higher in CAD patients than in controls. The rs330910T allele carriers in controls had lower serum low-density lipoprotein cholesterol (LDL-C) levels than the rs330910T allele non-carriers (*P* < 0.0014). The rs12785A, rs9949A and rs330910T allele carriers were associated with an increased risk of CAD (*P* = 0.008–0.004). There was strong linkage disequilibrium among the 4 SNPs in the controls and CAD/IS patients. The T-A-A-G haplotype was associated with a decreased risk of CAD and IS, whereas the A-A-T-A haplotype was associated with an increased risk for IS. Haplotype-environment interactions on the risk of CAD and IS were also observed.

**Conclusions:**

Several *PPP1R3B* polymorphisms were associated with serum LDL-C levels, the risk of CAD and IS in the Southern Chinese Han population. But these findings still need to be confirmed in the other populations with larger sample sizes.

**Electronic supplementary material:**

The online version of this article (10.1186/s12986-018-0266-y) contains supplementary material, which is available to authorized users.

## Background

The mortality and morbidity of coronary artery disease (CAD) and ischemic stroke (IS) remain very high in different districts [1–3]. Although both CAD and IS are complex multifactorial disorder, it is widely accepted that the pathological basis of the two diseases is atherosclerosis, and dyslipidemia plays an important role in process of coronary atherosclerosis [4]. Recently, Sabatine et al. [5] reported an inspiration result that a lipid-lowering drug, evolocumab (a monoclonal antibody) lowers low-density lipoprotein cholesterol (LDL-C) levels by approximately 60%. CAD and IS also share some common genetic and environmental determinants such as sex, age, obesity, cigarette smoking, hyperlipidemia, and hypertension [6–11]. Previous genome-wide association studies (GWASes) identified many genetic variants influenced the risk of CAD or IS [11] and it was reported that heritability estimates of coronary artery calcification are approximately 50% [12, 13].

The protein phosphatase 1 regulatory subunit 3B gene (*PPP1R3B*) is located on chromosome 8p. The gene which encoding a regulatory subunit of protein phosphatase 1 is involved in modulation of glycogen synthesis in liver and skeletal muscle [14–16]. Several previous studies have also found the association of variants in *PPP1R3B* locus with other cardiometabolic risk factors such as fasting glucose, fasting insulin, ferritin, liver enzymes, and nonalcoholic fatty liver disease [17–20]. Manning et al. [17] reported that variants in the *PPP1R3B* were associated with fasting glucose and fasting insulin at genome-wide levels of significance (*P* < 5 × 10^− 8^). Mehta et al. [18] found that *PPP1R3B* promoted hepatic glycogen synthesis and thereby regulated fasting energy homeostasis in mice those with liver-specific overexpression of PPP1R3B. Kahali et al. [19] showed that higher expression of variant falling in noncoding regions of *PPP1R3B* promoted nonalcoholic fatty liver disease and suggesting that this could be a functional expression quantitative trait locus (eQTL) variant [20]. Although the regulatory mechanism of the *PPP1R3B* expression is not well-known, it was acknowledged that the 3′-untraslated region plays an important role in governing spatial and temporal translation of a mRNA [21]. Two previous GWASes showed that single nucleotide polymorphisms (SNPs) in the *PPP1R3B* were associated with plasma lipid levels including high-density lipoprotein cholesterol (HDL-C), LDL-C, and total cholesterol (TC) and the risk of CAD [22, 23]. A study showed that higher expression of *PPP1R3B* by adenovirus administration would lower HDL-C levels in mouse liver [22]. In addition, a previous study in the Chinese Han population has showed that three *PPP1R3B* SNPs were significantly associated with plasma LDL-C (rs2126259, rs9987289 and rs19334) and TC (rs2126259 and rs9987289), and two SNPs with C-reactive protein (CRP; rs189798 and rs330919) but not with plasma lipid levels [24]. These findings suggest that the other SNPs in the 3’UTR of *PPP1R3B* may also influence serum lipid levels and the risk of CAD and IS. Therefore, the purpose of the present study was to detect the association of four novel *PPP1R3B* SNPs (rs12785, rs330910, rs330915 and rs9949) and their haplotypes with serum lipid levels and the risk of CAD and IS in a Southern Chinese Han population.

## Methods

### Study patients

The study samples contained 1087 unrelated patients (CAD, *n* = 556 and IS, *n* = 531). All of them were the hospitalized patients in the First Affiliated Hospital, Guangxi Medical University. The diagnosis of CAD based on typical clinical, discomfort, electrocardiographic changes, as well as cardiac makers (creatinine kinase-MB and troponin T). CAD was defined by coronary angiography which two independent angiographers were both blinded to the outcome of the genotypes. Coronary stenosis ≥50% in at least one of the three main coronary vessels was considered significant [25]. All of the IS patients received a strict neurological examination, and brain magnetic resonance imaging (MRI) was performed. The diagnosis of IS was according to TOAST (Trial of Org 10,712 in Acute Stroke Treatment) criteria, and patients included met one or two criteria: large-artery thrombosis and small-vessel occlusion [26]. All of the participants with a history of autoimmune, hematologic, neoplastic, liver, renal, thyroid, and type 1 diabetes were rejected. CAD subjects with a history of IS or IS patients with a history of CAD were excluded.

### Control subjects

A total of 617 control subjects matched by age, gender, ethnic group (Han Chinese in Guangxi, China) were also included. All of the individuals were randomly selected from the Physical Examination Center of the First Affiliated Hospital during the same period. Questionnaires, history-taking, strict clinical examination and image examinations (computed tomography or MRI) were used to insure all the participants free of CAD and IS. All participants have provided their written informed consents and the study protocol was approved by the Ethics Committee of the First Affiliated Hospital, Guangxi Medical University (No: Lunshen-2011-KY-Guoji-001; Mar. 7, 2011). The reported investigations were in accordance with the principles of the Declaration of Helsinki.

### Biochemical measurements

Before venous blood samples were obtained, all participants fasted at least 12 h. The levels of TC, triglyceride (TG), HDL-C, and LDL-C were determined by enzymatic methods with commercially available kits (RANDOX Laboratories). Serum apolipoprotein (Apo) A1 and ApoB levels were detected by the immunoturbidimetric immunoassay. The normal values in our Clinical Science Experiment Center were 3.10–5.17 mmol/L for TC, 0.56–1.70 mmol/L for TG, 0.91–1.81 mmol/L for HDL-C, 2.70–3.20 mmol/L for LDL-C, 1.00–1.78 g/L for ApoA1, 0.63–1.14 g/L for ApoB levels, and 1.00–2.50 for the ApoA1/ApoB ratio. The participants with TC > 5.17 mmol/L, and/or TG > 1.70 mmol/L were defined as hyperlipidemic [27, 28]. Hypertension was defined as a systolic blood pressure (SBP) of 140 mmHg or greater, and/or a diastolic blood pressure (DBP) of 90 mmHg or higher [29]. Drinking based on alcohol consumption (yes or no). Individuals’ age was divided into > 60- or ≤ 60-year subgroups. Body mass index (BMI) was calculated according to the values of weight divided by height squared (kg/m^2^). A BMI of ≤24, 24–28, and > 28 kg/m^2^ was defined as normal weight, overweight and obesity, respectively. Smoking was defined as current smoking (yes or no).

### SNPs selection

The selection of SNPs was according to the following assumption: (1) Variants in the *PPP1R3B* were proved to be associated with plasma lipid levels and cardiovascular disease according to previous GWASes [22, 30–32]. (2) Selected SNPs were established by Haploview (Broad Institute or MIT and Harvard, Cambridge, MA, USA, version 4.2). (3) Variant Effect Predictor from online resource (1000 Genome Project Database) predicted that selected SNPs participate in protein-coding and lead to serum lipid changes (Additional file 1 Figure. S1). (4) SNPs information was obtained from NCBI dbSNP Build 132 (http://www.Ncbi.nlm. nih.gov/SNP/). (5) SNPs were restricted to minor allele frequency (MAF) > 1%. (6) The *PPP1R3B* rs12785, rs330910, rs330915 and rs9949 SNPs were selected by the block-based approach. This strategy was enabled by the correlations between tagging SNPs manifested as linkage disequilibrium (LD). Although classic tagging is not the goal of SNP selection, with innovative tagging SNP selection bias is inevitable [33–37].

### Genotyping

Genomic DNA was extracted from leucocytes of venous blood using the phenol-chloroform method. Genotyping of the SNPs was accomplished by the Snapshot technology platform in the Center for Human Genetics Research, Shanghai Genesky Bio-Tech Co. Ltd. [27, 28, 38–42]. The restriction enzymes for the SNPs were SAP (Promega) and Exonuclease I (Epicentre), respectively. The forward and reverse primers were 5’-GGGGCAACCTGGGAAAGATTC-3′ and 5’-GCCTACACACTTCAGAGGGTGACA-3′ for rs12785; 5’-TTGCTTCCATTTGAGTTCGATTTATG-3′ and 5’-CCTCTCCCAGGTGGGTAACACTCT-3′ for rs330910; 5’-CGGCCCCAGAGGTCTCTTTTAC-3′ and 5’-TCAGAAAATGGTTTTATCGTGACTGTG-3′ for rs330915; and 5’-GGAAGATCCAGAAAATGGGCAGT-3′ and 5’-ACAATGTCAGAGTCAATGGGAGAATTT-3′ for rs9949 SNPs, respectively.

### Statistical analyses

The statistical software of SPSS22.0 (SPSS Inc., Chicago, IL, USA) was used to carry out the statistical analyses. Quantitative variables were expressed as mean ± standard deviation (serum TG levels were expressed as medians and interquartile ranges). Qualitative variables were expressed as percentage. Allele frequency was determined via direct counting, and the standard goodness-of-fit test was used for the testing of Handy-Weinberg equilibrium (HWE). Sex ratio and the genotype distribution were evaluated by a chi-square analysis. The student’s unpaired *t*-test was used to test the general characteristics between patients and controls. Analysis of covariance (ANCOVA) was used to test the association of genotypes and cardiometabolic traits such as serum lipid parameters, BMI, SBP, DBP and pulse pressure (PP). Bonferroni correction was employed for variants associated with the cardiometabolic traits, and a *P* < 0.0014 (0.05/5 × 7) for serum lipid parameters, and *P* < 0.0025 (0.05/5 × 4) for BMI, SBP, DBP and PP were considered statistical significant. The associations of genotypes and the risk of CAD and IS, also the gene-environment interactions on the risk of CAD and IS were tested by the unconditional logistic regression after gender, age, BMI, smoking, alcohol consumption, hypertension and hyperlipidemia were adjusted [6, 7, 27, 28]. The correlation risk was estimated by odds ratio (OR) and 95% confidence interval (95%CI). The pattern of pair-wise LD between the four SNPs was measured by *D’* and *r*^2^ using the SHEsis software [43]. A two-tailed *P* value less than 0.05 was considered statistically significant for the remaining variables.

## Results

### General characteristics of the participants

The general characteristics of the participants are presented in Table 1. The mean levels of BMI, SBP, PP, TC, TG and ApoB were higher but the values of HDL-C and LDL-C were lower in CAD patients than in controls (*P* < 0.05 for all). The values of BMI, SBP, PP, TC and TG were higher whereas those of DBP, HDL-C, LDL-C and ApoB were lower in IS patients than in controls (*P* < 0.05 for all).

**Table 1:** General characteristic of the participants

Characteristic	Control	CAD	IS	*P*_CAD_	*P*_IS_
Number	617	556	531		
Male/female	445/172	410/146	384/147	0.534	0.942
Age, year	62.57 ± 11.79	62.22 ± 10.56	62.78 ± 12.38	0.174	0.083
Body mass index, kg/m^2^	22.60 ± 2.82	23.86 ± 3.24	23.44 ± 3.51	0.002	0.000
Systolic blood pressure, mmHg	128.23 ± 19.06	133.18 ± 23.42	147.78 ± 21.92	0.000	0.007
Diastolic blood pressure, mmHg	80.59 ± 11.47	79.14 ± 13.40	83.82 ± 12.92	0.058	0.006
Pulse pressure, mmHg	47.63 ± 13.70	54.05 ± 16.95	63.96 ± 17.81	0.000	0.000
Cigarette smoking, n (%)	242(39.2)	240(43.2)	221(41.6)	0.170	0.409
Alcohol consumption, n (%)	193(31.3)	154(27.7)	150(28.2)	0.179	0.263
Total cholesterol, mmol/L	4.88 ± 1.06	4.51 ± 1.16	4.52 ± 1.14	0.007	0.035
Triglyceride, mmol/L	1.22 (0.80)	1.64 (1.08)	1.62 (1.06)	0.000	0.000
HDL-C, mmol/L	1.89 ± 0.49	1.14 ± 0.34	1.22 ± 0.40	0.000	0.000
LDL-C, mmol/L	2.72 ± 0.77	2.70 ± 0.99	2.68 ± 0.89	0.000	0.000
Apolipoprotein (Apo) A1, g/L	1.41 ± 0.25	1.02 ± 0.31	1.03 ± 0.22	0.983	0.051
ApoB, g/L	0.90 ± 0.21	0.91 ± 0.26	0.89 ± 0.25	0.000	0.000
ApoA1/ApoB	1.64 ± 0.52	1.38 ± 2.48	1.26 ± 0.60	0.043	0.791

*CAD* coronary artery disease, *IS* ischemic stroke, *HDL-C* high-density lipoprotein cholesterol, *LDL-C* low-denstity lipoprotein cholesterol. The value of triglyceride was presented as median (interquartile tange), the difference between CAD/IS patients and controls was determined by the Wilcoxon-Mann-Whitney test

### Genotypic and allelic frequencies in controls and patients

The genotypic and allelic frequencies of the four *PPP1R3B* SNPs are summarized in Additional file 1 Table S1. The genotype distribution of the four SNPs was consistent with the HWE in patients and controls (*P*_HWE_ > 0.05 for all). The frequency of the rs12785A and rs9949A alleles was higher in CAD/IS patients than in controls (*P* < 0.05 for all). The frequency of the rs330910T and rs330915T alleles was also higher in CAD patients than in controls, but no difference was found between IS patients and controls. There was no difference in the genotype frequencies of the four SNPs between CAD/IS patients and controls.

### Genotypes and cardiometabolic traits

The associations of the four *PPP1R3B* SNPs and cardiometabolic traits such as serum lipid parameters, BMI, SBP, DBP and PP in controls are presented in Table 2 and Additional file 1 Table S2. The rs330910T allele carriers had lower serum LDL-C levels than the rs330910T allele non-carriers (rs330910AA homozygotes; *P* < 0.0014). There were no differences in serum TC, TG, HDL-C, ApoA1, ApoB levels, and the ApoA1/ApoB ratio among the genotypes of the remaining 3 SNPs. There were also no significant associations between the four SNPs and BMI, SBP, DBP and PP (*P* > 0.0025 for all; Additional file 1 Table S2).

**Table 2:** Association of the *PPP1R3B* genotypes and serum lipid levels in controls

Genotype	n	TC (mmol/L)	TG (mmol/L)	HDL-C (mmol/L)	LDL-C (mmol/L)	ApoA1 (g/L)	ApoB (g/L)	ApoA1/ ApoB
rs12785								
TT	356	4.88 ± 1.02	1.25 (0.89)	1.89 ± 0.48	2.79 ± 0.80	1.41 ± 0.26	0.90 ± 0.22	1.66 ± 0.56
TA	222	4.89 ± 0.87	1.21 (0.68)	1.92 ± 0.49	2.69 ± 0.72	1.4 1 ± 0.23	0.91 ± 0.23	1.64 ± 0.47
AA	39	4.47 ± 1.05	1.05 (0.82)	1.87 ± 0.52	2.41 ± 0.71	1.34 ± 0.30	0.88 ± 0.24	1.59 ± 0.41
*P*		0.036	0.320	0.623	0.010	0.231	0.798	0.714
TT	356	4.88 ± 1.02	1.25 (0.89)	1.89 ± 0.48	2.79 ± 0.80	1.41 ± 0.26	0.90 ± 0.22	1.66 ± 0.56
TA + AA	261	4.83 ± 0.91	1.19 (0.70)	1.92 ± 0.50	2.65 ± 0.81	1.40 ± 0.24	0.90 ± 0.23	1.63 ± 0.46
*P*		0.036	0.119	0.610	0.011	0.398	0.560	0.089
rs330910								
AA	396	4.86 ± 1.04	1.26 (0.87)	1.87 ± 0.48	2.77 ± 0.83	1.41 ± 0.26	0.91 ± 0.23	1.65 ± 0.55
AT	194	4.88 ± 0.86	1.18 (0.62)	1.95 ± 0.51	2.67 ± 0.66	1.42 ± 0.24	0.90 ± 0.22	1.66 ± 0.46
TT	27	4.64 ± 0.85	1.14 (0.95)	1.92 ± 0.43	2.50 ± 0.59	1.34 ± 0.19	0.93 ± 0.21	1.52 ± 0.40
*P*		0.522	0.492	0.171	0.092	0.381	0.730	0.416
AA	396	4.86 ± 1.04	1.26 (0.87)	1.87 ± 0.48	2.77 ± 0.83	1.41 ± 0.26	0.91 ± 0.23	1.65 ± 0.55
AT+TT	221	4.84 ± 0.86	1.17 (0.67)	1.95 ± 0.50	2.64 ± 0.65	1.41 ± 0.23	0.90 ± 0.22	1.64 ± 0.45
*P*		0.014	0.064	0.383	0.000	0.299	0.518	0.170
rs330915								
AA	409	4.89 ± 1.00	1.25 (0.85)	1.90 ± 0.48	2.77 ± 0.78	1.42 ± 0.26	0.90 ± 0.22	1.66 ± 0.54
AT	187	4.86 ± 0.87	1.22 (0.73)	1.92 ± 0.48	2.70 ± 0.74	1.40 ± 0.22	0.91 ± 0.26	1.62 ± 0.48
TT	21	4.16 ± 1.19	0.85 (0.37)	1.78 ± 0.59	2.24 ± 0.79	1.30 ± 0.37	0.80 ± 0.22	1.65 ± 0.38
*P*		0.003	0.080	0.488	0.009	0.119	0.095	0.765
AA	409	4.89 ± 1.00	1.25 (0.85)	1.90 ± 0.48	2.77 ± 0.78	1.42 ± 0.26	0.90 ± 0.22	1.66 ± 0.54
AT+TT	208	4.79 ± 0.93	1.18 (0.71)	1.91 ± 0.50	2.65 ± 0.75	1.39 ± 0.24	0.90 ± 0.25	1.63 ± 0.47
*P*		0.172	0.232	0.753	0.325	0.531	0.744	0.266
rs9949								
GG	357	4.87 ± 1.02	1.25 (0.87)	1.89 ± 0.48	2.79 ± 0.80	1.41 ± 0.26	0.90 ± 0.22	1.66 ± 0.55
GA	222	4.89 ± 0.87	1.22 (0.68)	1.92 ± 0.49	2.69 ± 0.72	1.41 ± 0.23	0.91 ± 0.23	1.64 ± 0.47
AA	38	4.47 ± 1.07	1.06 (0.80)	1.88 ± 0.53	2.40 ± 0.72	1.34 ± 0.30	0.88 ± 0.25	1.58 ± 0.41
*P*		0.043	0.354	0.695	0.010	0.182	0.783	0.705
GG	357	4.87 ± 1.02	1.25 (0.87)	1.89 ± 0.48	2.79 ± 0.80	1.41 ± 0.26	0.90 ± 0.22	1.66 ± 0.55
GA + AA	260	4.82 ± 0.91	1.19 (0.70)	1.01 ± 0.50	2.65 ± 0.73	1.40 ± 0.24	0.90 ± 0.23	1.63 ± 0.46
*P*		0.044	0.126	0.585	0.011	0.401	0.594	0.104

*TC* total cholesterol, *TG* triglyceride, *HDL-C* high-density lipoprotein cholesterol, *LDL-C* low-denstity lipoprotein cholesterol, *ApoA1* apolipoprotein A1, *ApoB* apolipoprotein B. The value of triglyceride was presented as median (interquartile range), and the difference among or between the genotypes was determined by the Kruskal-Wallis test or Wilcoxon-Mann-Whitney test. A *P* < 0.0014 was considered statistically significant after Bonferroni correction

### *PPP1R3B* genotypes and the risk of CAD and IS

The associations of the four *PPP1R3B* SNPs and the risk of CAD and IS are shown in Table 3. After controlling for potential confounders including gender, sex, BMI, cigarette smoking, alcohol consumption, hypertension and hyperlipidemia, the genotypes of rs12785, rs330910 and rs9949 SNPs were associated with the risk of CAD (*P* = 0.008–0.004). The subjects with rs12785TA/AA genotypes (Dominant: OR = 1.32, 95%CI = 1.02–1.71, *P* = 0.009; Log-additive: OR = 1.25, 95%CI = 1.02–1.54, *P* = 0.007); rs330910AT/TT genotypes (Dominant: OR = 1.35, 95%CI = 1.04–1.76, *P* = 0.006; Log-additive: OR = 1.25, 95%CI = 1.04–1.60, *P* = 0.004); and rs9949GA/AA genotypes (Dominant: OR = 1.31, 95%CI = 1.02–1.73, *P* = 0.010; Log-additive: OR = 1.25, 95%CI = 1.02–1.53, *P* = 0.008) had higher risk of CAD, respectively. No significant association between the four SNPs and the risk of IS was observed.

**Table 3:** Association between the *PPP1R3B* polymorphisms and the risk of CAD and IS

SNP/Model	Genotype	OR (95%CI)_CAD_	*P*_CAD_	OR (95%CI)_IS_	*P*_IS_
rs12785					
Dominant	TT	1.00		1.00	
	TA + AA	1.32(1.02–1.71)	0.009	1.12(0.86–1.46)	0.405
Log-additive	–	1.25(1.02–1.54)	0.007	1.16(0.94–1.42)	0.178
rs330910					
Dominant	AA	1.00		1.00	
	AT+TT	1.35(1.04–1.76)	0.006	1.03(0.79–1.36)	0.812
Log-additive	–	1.29(1.04–1.60)	0.004	1.08(0.87–1.35)	0.489
rs330915					
Dominant	AA	1.00		1.00	
	AT+TT	1.15(0.88–1.51)	0.291	1.15(0.87–1.51)	0.330
Log-additive	–	1.19(0.95–1.49)	0.122	1.16(0.92–1.47)	0.218
rs9949					
Dominant	GG	1.00		1.00	
	GA + AA	1.31(1.02–1.73)	0.010	1.14(0.87–1.48)	0.341
Log-additive	–	1.25(1.02–1.53)	0.008	1.17(0.95–1.44)	0.137

*SNP* single nucleotide polymorphisms *CAD* coronary artery disease *IS* ischemic disease

### LD analyses

**Fig. 1:**
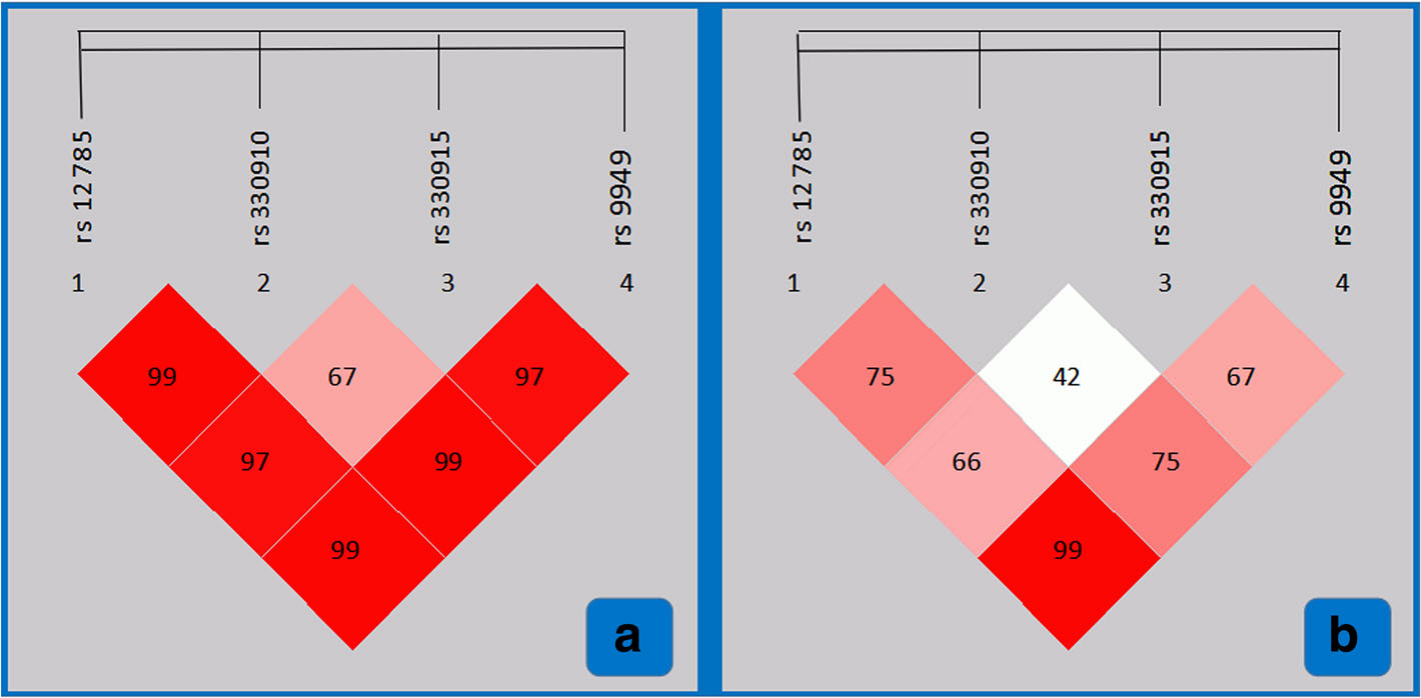
The linkage disequilibrium (LD) analyses among the four *PPP1R3B* SNPs of rs12785, rs330910, rs330915 and rs9949. (**a**) *D’*; (**b**) *r*^2^

As shown in Additional file 1 Table S3 and Fig. 1, there was strong LD among the rs12785, rs330910, rs330915 and rs9949 SNPs in the controls and CAD/IS patients. The LD among the rs12785, rs9949 and rs330910 was strong (*D’* = 0.997–0.998, *r*^2^ = 0.751–0.991). The LD among the rs12785, rs9949 and rs330915 was also strong (*D’* = 0.975–0.998, *r*^2^ = 0.669–0.991). The SNPs of rs12785 and 9949 almost reached a complete LD (*r*^2^ = 0.993), whereas the LD between the rs330910 and rs330915 SNPs was weak (*D’* = 0.673).

### Haplotypes and the risk of CAD and IS

As shown in Additional file 1 Table S4, the commonest haplotype was T-A-A-G (in the order of rs12785-rs330910-rs330915-rs9949), it represented more than 70% of the participants. The haplotype of T-A-A-G was also associated with a reduced risk for CAD (*P =* 0.014) and IS (*P =* 0.023). The haplotype of A-A-T-A was associated with an increased risk for IS (*P =* 0.036).

### Gene-environment interactions on the risk of CAD and IS

As shown in Table 4, there were no significant gene-environment interactions after Bonferroni correction in this study (*P* > 0.0017 for all).

**Table 4:** The *PPP1R3B* SNP-environment interactions on the risk of CAD and IS

Factor	rs12785		rs330910		rs330915		rs9949	
	*P*_CAD_	*P*_IS_	*P*_CAD_	*P*_IS_	*P*_CAD_	*P*_IS_	*P*_CAD_	*P*_IS_
Sex (female vs. male)	0.015	0.80	0.006	0.30	0.79	0.45	0.017	0.80
Age (≤ 60 vs. > 60 year)	0.84	0.41	0.75	0.12	0.50	0.71	0.85	0.42
BMI (≤ 24 vs. > 24 kg/m^2^)	0.89	0.12	0.78	0.65	0.78	0.48	0.92	0.12
Smoking (yes vs. no)	0.64	0.010	0.57	0.15	0.29	0.026	0.62	0.010
Drinking (yes vs. no)	0.61	0.92	0.41	0.41	0.62	0.87	0.57	0.96
hypertension (yes vs. no)	0.009	0.22	0.007	0.77	0.014	0.51	0.009	0.23
hyperlipidemia (yes vs. no)	0.25	0.12	0.32	0.092	0.28	0.37	0.24	0.14

*P*_CAD_/*P*_IS,_ the *P* value between patients and control, *BMI* body mass index. The *P* values for interactions of genotypes and gender, age, BMI, drinking, smoking, hypertension, hyperlipidemia on the risk of CAD/IS were obtained from unconditional logistic regression, and a *P* < 0.0017 was considered statistically significant after Bonferroni correction

### Stratified analyses of the *PPP1R3B* SNPs on the risk of CAD and IS

As described in Table 5, stratified analysis between the *PPP1R3B* polymorphisms and the risk of CAD and IS in dominant model showed that the rs12785A (OR = 1.99, 95% CI = 1.28–3.12), rs330910T (OR = 2.07, 95% CI = 1.32–3.26) and rs9949A allele (OR = 1.74, 95% CI = 1.12–2.71) carriers in females were associated with an increased risk of CAD compared with their own homozygotes (rs12785TT/rs330910AA/rs9949GG carriers); the rs12785A (OR = 1.82, 95% CI = 1.25–2.69), rs330910T (OR = 1.90, 95% CI = 1.29–2.80), rs330915T (OR = 1.69, 95% CI = 1.14–2.49); and rs9949A allele (OR = 1.82, 95% CI = 1.25–2.66) carriers in normotensives were associated with an increased risk of CAD compared with their own homozygotes (rs12785TT/rs330910AA/rs330915AA/rs9949GG carriers); and the rs12785A (OR = 1.57, 95% CI = 1.05–2.40) and rs9949A allele (OR = 1.56, 95% CI = 1.06–2.39) carriers in smokers were associated with an increased risk of IS compared with their own homozygotes (rs12785TT/rs9949GG carriers).

**Table 5:** Stratified analysis of the *PPP1R3B* genotypes and the risk of CAD or IS

Factor	Genotype	OR(95%CI)_CAD_	*P*_CAD_	OR(95%CI)_IS_	*P*_IS_
rs12785					
Gender					
Male	TT	1		1	
	TA + AA	1.06(0.68–1.44)	0.830	1.15(0.80–1.56)	0.470
Female	TT	1		1	
	TA + AA	1.76(1.13–2.73)	0.007	1.00(0.63–1.56)	0.970
Age, year					
≤ 60	TT	1		1	
	TA + AA	1.34(0.73–1.89)	0.280	1.03(0.70–1.50)	0.980
> 60	TT	1		1	
	TA + AA	1.24(0.85–1.82)	0.260	1.23(0.84–1.80)	0.280
BMI, kg/m^2^					
≤ 24	TT	1		1	
	TA + AA	1.27(0.93–1.74)	0.970	1.25(0.91–1.71)	0.170
> 24	TT	1		1	
	TA + AA	1.23(0.79–1.91)	0.370	0.80(0.49–1.32)	0.400
Smoking					
No	TT	1		1	
	TA + AA	1.34(0.97–1.87)	0.090	0.85(0.60–1.19)	0.330
Yes	TT	1		1	
	TA + AA	1.15(0.78–1.71)	0.520	1.56(1.05–2.39)	0.009
Drinking					
No	TT	1		1	
	TA + AA	1.19(0.88–1.63)	0.270	1.06(0.77–1.45)	0.720
Yes	TT	1		1	
	TA + AA	1.46(0.93–2.27)	0.100	1.03(0.66–1.61)	0.910
Hyperlipidemia					
No	TT	1		1	
	TA + AA	1.47(1.05–2.08)	0.090	0.92(0.66–1.32)	0.700
Yes	TT	1		1	
	TA + AA	1.07(0.71–1.60)	0.740	1.39(0.94–2.05)	0.100
Hypertension					
No	TT	1		1	
	TA + AA	1.82(1.25–2.69)	0.002	1.28(0.91–1.80)	0.160
Yes	TT	1		1	
	TA + AA	0.94(0.66–1.34)	0.750	0.92(0.59–1.34)	0.610
rs330910					
Gender					
Male	AA	1		1	
	AT+TT	0.99(0.72–1.36)	0.960	0.91(0.66–1.27)	0.580
Female	AA	1		1	
	AT+TT	2.07(1.32–3.26)	0.002	1.16(0.73–1.85)	0.530
Age, year					
≤ 60	AA	1		1	
	AT+TT	1.33(0.94–1.87)	0.110	0.84(0.58–1.21)	0.350
> 60	AA	1		1	
	AT+TT	1.24(0.84–1.84)	0.280	1.27(0.86–1.88)	0.220
BMI, kg/m^2^					
≤ 24	AA	1		1	
	AT+TT	1.25(0.91–1.72)	0.170	1.04(0.75–1.43)	0.820
> 24	AA	1		1	
	AT+TT	1.30(0.83–2.06)	0.260	0.91(0.57–1.46)	0.690
Smoking					
No	AA	1		1	
	AT+TT	1.37(0.97–1.92)	0.070	0.85(0.60–1.20)	0.360
Yes	AA	1		1	
	AT+TT	1.16(0.77–1.74)	0.470	1.21(0.79–1.86)	0.390
Drinking					
No	AA	1		1	
	AT+TT	1.18(0.86–1.62)	0.300	1.06(0.76–1.48)	0.720
Yes	AA	1		1	
	AT+TT	1.01(0.74–1.38)	0.960	0.82(0.52–1.30)	0.390
Hyperlipidemia					
No	AA	1		1	
	AT+TT	1.48(1.04–2.10)	0.030	0.81(0.56–1.16)	0.250
Yes	AA	1		1	
	AT+TT	1.10(0.73–1.64)	0.650	1.30(0.87–1.93)	0.280
Hypertension					
No	AA	1		1	
	AT+TT	1.90(1.29–2.80)	0.001	1.05(0.74–1.49)	0.770
Yes	AA	1		1	
	AT+TT	0.93(0.64–1.34)	0.680	0.94(0.62–1.42)	0.770
rs330915					
Gender					
Male	AA	1		1	
	AT+TT	1.20(0.87–1.66)	0.270	1.22(0.87–1.70)	0.250
Female	AA	1		1	
	AT+TT	1.12(0.71–1.77)	0.610	0.89(0.56–1.42)	0.630
Age, year					
≤ 60	AA	1		1	
	AT+TT	1.20(0.87–1.66)	0.270	1.09(0.75–1.57)	0.670
> 60	AA	1		1	
	AT+TT	1.30(0.88–1.94)	0.190	1.18(0.79–1.75)	0.410
BMI, kg/m^2^					
≤ 24	AA	1		1	
	AT+TT	1.19(0.86–1.65)	0.290	1.20(0.86–1.66)	0.280
> 24	AA	1		1	
	AT+TT	1.09(0.69–1.73)	0.700	0.98(0.61–1.57)	0.940
Smoking					
No	AA	1		1	
	AT+TT	1.31(0.93–1.84)	0.130	0.89(0.63–1.25)	0.510
Yes	AA	1		1	
	AT+TT	0.97(0.64–1.48)	0.910	1.54(0.99–2.40)	0.060
Drinking					
No	AA	1		1	
	AT+TT	1.21(0.88–1.68)	0.240	1.09(0.78–1.53)	0.610
Yes	AA	1		1	
	AT+TT	1.10(0.69–1.76)	0.680	1.04(0.66–1.65)	0.870
Hyperlipidemia					
No	AA	1		1	
	AT+TT	1.37(0.96–1.96)	0.070	1.03(0.72–1.48)	0.870
Yes	AA	1		1	
	AT+TT	1.04(0.69–1.57)	0.860	1.31(0.87–1.97)	0.190
Hypertension					
No	AA	1		1	
	AT+TT	1.69(1.14–2.49)	0.008	1.24(0.87–1.77)	0.240
Yes	AA	1		1	
	AT+TT	0.89(0.61–1.30)	0.530	1.00(0.66–1.51)	0.990
rs9949					
Gender					
Male	GG	1		1	
	GA + AA	1.07(0.79–1.46)	0.660	1.13(0.82–1.55)	0.470
Female	GG	1		1	
	GA + AA	1.74(1.12–2.71)	0.008	1.00(0.63–1.56)	0.980
Age, year					
≤ 60	GG	1		1	
	GA + AA	1.28(0.91–1.80)	0.150	1.03(0.72–1.47)	0.960
> 60	GG	1		1	
	GA + AA	1.24(0.85–1.82)	0.260	1.24(0.84–1.81)	0.280
BMI, kg/m^2^					
≤ 24	GG	1		1	
	GA + AA	1.27(0.93–1.73)	0.960	1.25(0.91–1.71)	0.170
> 24	GG	1		1	
	GA + AA	1.23(0.79–1.92)	0.37	0.82(0.52–1.31)	0.410
Smoking					
No	GG	1		1	
	GA + AA	1.34(0.96–1.86)	0.086	0.85(0.60–1.19)	0.330
Yes	GG	1		1	
	GA + AA	1.15(0.77–1.71)	0.520	1.56(1.06–2.39)	0.009
Drinking					
No	GG	1		1	
	GA + AA	1.190.88–1.63)	0.260	1.06(0.77–1.46)	0.720
Yes	GG	1		1	
	GA + AA	1.46(0.93–2.27)	0.100	1.03(0.66–1.61)	0.910
Hyperlipidemia					
No	GG	1		1	
	GA + AA	1.48(1.05–2.09)	0.030	0.93(0.66–1.33)	0.700
Yes	GG	1		1	
	GA + AA	1.07(0.72–1.59)	0.740	1.39(0.94–2.05)	0.100
Hypertension					
No	GG	1		1	
	GA + AA	1.82(1.25–2.66)	0.002	1.28(0.91–1.80)	0.160
Yes	GG	1		1	
	GA + AA	0.94(0.66–1.35)	0.750	0.91(0.61–1.35)	0.630

*P*, the *P* value between patients and control, *BMI* body mass index. The *P* values for stratified analysis between the *PPP1R3B* polymorphisms on the risk of CAD/IS, were obtained from unconditional logistic regression, when one of the environmental factors was detected, the rest six factors in gender, age, BMI, smoking, alcohol consumption, hypertension and hyperlipidemia were adjusted

### Haplotype-environment interactions on the risk of CAD and IS

The interactions of several haplotypes and envirnmental factors on the risk of CAD were also noted in this study. When one of the environmental factors was detected, the rest six factors of gender, age, BMI, smoking, alcohol consumption, hypertension and hyperlipidemia were adjusted.

For the interactions of haplotype-gender on the risk of CAD, the haplotypes of A-T-T-A (OR = 1.70, 95% CI = 1.09–2.65) and A-T-A-A (OR = 3.49, 95%CI = 1.70–7.17) in females were associated with an increased risk of CAD as compared to the T-A-A-G haplotypes (interacted *P*-value = 0.018). As compared with the same haplotype, the haplotypes of T-A-A-G (OR = 3.02, 95%CI = 2.07–4.39), A-T-T-A (OR = 2.07, 95%CI = 1.32–3.26), and A-A-T-A (OR = 4.09, 95%CI = 1.62–9.31) in males were associated with an increased risk for CAD.

For the interactions of the haplotype-hypertension on the risk of CAD, the haplotype of A-T-T-A (OR = 1.68, 95% CI = 1.18–2.39) in normotensives was associated with an increased risk for CAD compared to the T-A-A-G haplotype. As compared with the haplotype in normotensives, the haplotypes of T-A-A-G (OR = 0.61, 95% CI = 0.42–0.88) and A-T-T-A (OR = 0.35, 95% CI = 0.22–0.55) in hypertension participants were associated with a decreased risk for CAD.

## Discussion

Previous study has showed that the *PPP1RB* polymorphisms were associated with hepatic steatosis [26] and plasma serum CRP traits [24, 44]. Ligthart et al. [45] reported that the *PPP1R3B* regions had a pleiotropic effect on CRP independent of the effects on the cardiometabolic phenotypes. Two previous GWASes showed that the *PPP1R3B* polymorphisms were associated with serum lipid traits, and higher expression of PPP1R3B would influence the lipid traits in animal studies [22, 23]. Furthermore, the *PPP1R3B* variants were found to influence cardiovascular events and atherosclerosis. Zhang et al. [24] reported that the *PPP1R3B* polymorphisms were associated with LDL-C and serum CRP levels. In a recent study, however, López-Mejías et al. [46] reported that the *PPP1R3B* SNPs were not associated with cardiovascular disease in rheumatoid arthritis patients. In the present study, we firstly disclose that the genetic polymorphisms in the 3’UTR of *PPP1R3B* were associated with serum LDL-C levels, the risk of CAD and IS in a Southern Chinese Han population.

In the present study, we showed that the A and T allele frequencies of rs12785 in controls were 24% and 76%; the A and G allele frequencies of rs9949 were 24% and 76%; the T and A allele frequencies of rs330910 were 20% and 80%; and the T and A allele frequencies of rs330915 were 19% and 81%, respectively. However, the data from the International HapMap project showed that the allele frequencies of the four SNPs were different. For SNPs of rs12785 and rs9949, which were in complete LD in CHB (Han Chinese in Beijing, China) and CEU (Utah residents with ancestry from northern and western Europe) both with an *r*^2^ = 1, in strong LD in CLM (Colombians from Medellin, Colombia) with an *r*^2^ = 0.992. The A and T allele frequencies of rs12785 were 29.6% and 70.4% in CHB (A < T); 76.8% and 23.2 in CEU (A > T); 65.9% and 34.1% in CLM (A < T). The A and G allele frequencies of rs9949 were 29.6% and 70.4% in CHB (A < G), 76.8% and 23.2% in CEU (A > G), 65.4% and 34.6% in CLM (A > G). For the rs330910 SNP, the T and A allele frequencies of rs330910 were 21.4% and 78.6% in CHB (T < A), 76.3% and 23.7% in CEU (T > A), and 55.4% and 44.6% in ITU (Indian Telugu from the UK). For the rs330915 SNP, meanwhile, the T and A allele frequencies were different among controls in our study, CHB, CEU and CLM. The allele frequencies were different between case and control in our research, just like rs330915 associated with CAD, rs12785/rs9949 associated with IS, even though it confirmed that the allele might not be associated with the risk of diseases after controlling potential confounders. These differences might due to racial-specificity and indicated that variants in the 3’UTR of *PPP1R3B* interacted with environment factors on the risk of diseases. More and larger samples of epidemiological investigation are necessary to confirm the interactions.

The results of our present study showed that serum LDL-C levels were different among the three genotypes of the rs330910 SNP. The rs330910T allele carriers in controls had lower serum LDL-C levels than the rs330910T allele non-carriers (*P* < 0.0014). In a previous GWAS, Teslovich et al. showed that the SNP in *PPP1R3B* was associated with HDL-C, TC and LDL-C [22]. But in our current study, no significant association was found in TC and HDL-C among the *PPP1R3B* genotypes. These findings might due to SNP chosen in GWAS located in different functional consequence and different sample sizes. Thus, the association between the *PPP1R3B* SNPs and serum lipid levels need to be confirmed by proteomics study in further research.

In the current study, we firstly showed that the rs12785, rs330910 and rs9949 SNPs were associated with CAD but not with IS. Haplotype analysis of the four SNPs showed that T-A-A-G was associated with a decreased risk for CAD and IS, and A-A-T-A was associated with an increased risk for IS. Due to rs12785 and rs9949 SNPs were in complete LD, the results of two SNPs were exactly similar to each other. The interactions of the *PPP1R3B* haplotypes and some environmental factors on the risk of CAD and IS remain unknown. In the present study, we firstly found that the haplotypes of A-T-T-A (OR = 1.70, 95% CI = 1.09–2.65) and A-T-A-A (OR = 3.49, 95%CI = 1.70–7.17) were associated with an increased risk of CAD. The haplotype of A-T-T-A (OR = 1.68, 95% CI = 1.18–2.39) was associated with an increased risk of CAD in normotensives. However, these findings need to be confirmed in the other populations with larger sample sizes.

Several potential limitations should be pointed out in this study. Firstly, the sample size was relatively small compared to many GWASes and molecular epidemiological investigations. Therefore, larger sample sizes are needed to confirm our results. Secondly, although several confounders have been adjusted for the statistical analyses in this study, individuals exposed to other genetic and environmental risk factors probably modify the association of genetic polymorphisms and risk of CAD and IS. Thirdly, we could not analyze the association of the *PPP1R3B* SNPs and serum lipid levels in the CAD/IS patients because of the influence of lipid-lowering drugs. Finally, it is well known that both CAD and IS are complex multifactorial disorder. Although we have detected the association of four *PPP1R3B* SNPs and their haplotypes with serum lipid levels and the risk of CAD and IS in this study, there are still many unmeasured genetic and environmental factors and their interactions. Thus, our findings still need to be confirmed in the other populations with larger sample sizes.

## Conclusions

Our present study suggests that the SNPs of rs12785, rs330910, and rs9949 were associated with the risk of CAD. The rs330910 SNP was associated with LDL-C levels. The rs330910T allele carriers in controls had lower LDL-C levels than the homozygote of rs330910AA. The carriers of rs12785A, rs330910T, rs9949A were associated with increased risk of CAD. The haplotype of T-A-A-G was associated with decreased risk of CAD and IS, and haplotype of A-A-T-A was associated with an increased risk of IS. These results suggest that the detected *PPP1R3B* SNPs were associated with serum LDL-C levels and the risk of CAD and IS in the Southern Chinese Han population. Detection of these *PPP1R3B* SNPs in our study population may have important significance for early diagnosis and future individualized treatment of dyslipidemia, CAD and IS.

## Additional file

Additional file 1: **Table S1.** Genotypic and allelic frequencies of the *PPP1R3B* polymorphisms in control and patients [n (%)]. **Table S2.** Association between the *PPP1R3B* genotypes and BMI or blood pressure in controls. **Table S3.** Linkage disequilibrium analysis of the 4 *PPP1R3B* SNPs. **Table S4.** Haplotype analysis of the 4 *PPP1R3B* SNPs. **Figure S1.** Annotation of SNPs in UCSC. Non-coding (ncRNA) and untranslated region (3’UTR) are colored blue. (DOC 392 kb)

## Abbreviations

ANCOVA: Analysis of covariance
Apo: Apolipoprotein
BMI: Body mass index
CAD: Coronary artery disease
CEU: Utah residents with ancestry from northern and western Europe
CHB: Han Chinese in Beijing, China
CI: Confidence interval
CLM: Colombians from Medellin, Colombia
CRP: C-reactive protein
DBP: Diastolic blood pressure
GWAS: Genome-wide association study
HDL-C: High-density lipoprotein cholesterol
HWE: Handy-Weinberg equilibrium
IS: Ischemic stroke
LD: Linkage disequiblirium
LDL-C: Low-density lipoprotein cholesterol
MAF: Minor allele frequency
MRI: Magnetic resonance imaging
OR: Odds ratio
PP: Pulse pressure
PPP1R3B: Protein phosphatase 1 regulatory subunit 3B
SBP: Systolic blood pressure
SNPs: Single nucleotide polymorphisms
TC: Total cholesterol
TG: Triglyceride
TOAST: Trial of Org 10,712 in Acute Stroke Treatment

## References

[1] Bhatnagar P, Wickramasinghe K, Wilkins E, Townsend N (2016). Trends in the epidemiology of cardiovascular disease in the UK. Heart.

[2] Saito I, Yamagishi K, Kokubo Y, Yatsuya H, Iso H, Sawada N (2016). Association between mortality and incidence rates of coronary heart disease and stroke: the Japan public health center-based prospective (JPHC) study. Int J Cardiol.

[3] Doherty JU, Kort S, Mehran R, Schoenhagen P, Soman P, Writing Group Members (2016). Executive summary: heart disease and stroke Statistics-2016 update a report from the American Heart Association. Circulation.

[4] Chhatriwalla AK, Nicholls SJ, Wang TH, Wolski K, Sipahi I, Crowe T (2009). Low levels of low-density lipoprotein cholesterol and blood pressure and progression of coronary atherosclerosis. J Am Coll Cardiol.

[5] Sabatine MS, Giugliano RP, Keech AC, Honarpour N, Wiviott SD, Murphy SA (2017). Evolocumab and clinical outcomes in patients with cardiovascular disease. N Engl J Med.

[6] Catapano AL, Reiner Z, De Backer G, Graham I, Taskinen MR, Wiklund O (2011). ESC/EAS guidelines for the management of dyslipidaemias the task force for the management of dyslipidaemias of the European Society of Cardiology (ESC) and the European atherosclerosis society (EAS). Atherosclerosis.

[7] Goldstein LB, Bushnell CD, Adams RJ, Appel LJ, Braun LT, Chaturvedi S (2011). Guidelines for the primary prevention of stroke: a guideline for healthcare professionals from the American Heart Association/American Stroke Association. Stroke.

[8] Klatsky AL (2015). Alcohol and cardiovascular diseases: where do we stand today?. J Intern Med.

[9] Nakanishi R, Berman DS, Budoff MJ, Gransar H, Achenbach S, Al-Mallah M (2015). Current but not past smoking increases the risk of cardiac events: insights from coronary computed tomographic angiography. Eur Heart J.

[10] Smith CY, Bailey KR, Emerson JA, Nemetz PN, Roger VL, Palumbo PJ (2015). Contributions of increasing obesity and diabetes to slowing decline in subclinical coronary artery disease. J Am Heart Assoc.

[11] Dichgans M, Malik R, König IR, Rosand J, Clarke R, Gretarsdottir S (2014). Shared genetic susceptibility to ischemic stroke and coronary artery disease: a genome-wide analysis of common variants. Stroke.

[12] Fischer M, Broeckel U, Holmer S, Baessler A, Hengstenberg C, Mayer B (2005). Distinct heritable patterns of angiographic coronary artery disease in families with myocardial infarction. Circulation.

[13] Vargas JD, Manichaikul A, Wang XQ, Rich SS, Rotter JI, Post WS (2016). Detailed analysis of association between common single nucleotide polymorphisms and subclinical atherosclerosis: the multi-ethnic study of atherosclerosis. Data Brief.

[14] Doherty MJ, Moorhead G, Morrice N, Cohen P, Cohen PT (1995). Amino acid sequence and expression of the hepatic glycogen-binding (GL)-subunit of protein phosphatase-1. FEBS Lett.

[15] Gasa R, Jensen PB, Berman HK, Brady MJ, DePaoli-Roach AA, Newgard CB (2000). Distinctive regulatory and metabolic properties of glycogen-targeting subunits of protein phosphatase-1 (PTG, GL, GM/RGl) expressed in hepatocytes. J Biol Chem.

[16] Munro S, Cuthbertson DJ, Cunningham J, Sales M, Cohen PT (2002). Human skeletal muscle expresses a glycogen-targeting subunit of PP1 that is identical to the insulin-sensitive glycogen-targeting subunit G(L) of liver. Diabetes.

[17] Manning AK, Hivert MF, Scott RA, Grimsby JL, Bouatia-Naji N, Chen H (2012). A genome-wide approach accounting for body mass index identifies genetic variants influencing fasting glycemic traits and insulin resistance. Nat Genet.

[18] Mehta MB, Shewale SV, Sequeira RN, Millar JS, Hand NJ, Rader DJ (2017). Hepatic protein phosphatase 1 regulatory subunit 3B (Ppp1r3b) promotes hepatic glycogen synthesis and thereby regulates fasting energy homeostasis. J Biol Chem.

[19] Kahali B, Halligan B, Speliotes EK (2015). Insights from genome-wide association analyses of nonalcoholic fatty liver disease. Semin Liver Dis.

[20] Speliotes EK, Yerges-Armstrong LM, Wu J, Hernaez R, Kim LJ, Palmer CD, Gudnason V (2011). Genome-wide association analysis identifies variants associated with nonalcoholic fatty liver disease that have distinct effects on metabolic traits. PLoS Genet.

[21] Kuersten S, Goodwin EB (2003). The power of the 3' UTR: translational control and development. Nat Rev Genet.

[22] Teslovich TM, Musunuru K, Smith AV, Edmondson AC, Stylianou IM, Koseki M (2010). Biological, clinical and population relevance of 95 loci for blood lipids. Nature.

[23] Waterworth DM, Ricketts SL, Song K, Chen L, Zhao JH, Ripatti S (2010). Genetic variants influencing circulating lipid levels and risk of coronary artery disease. Arterioscler Thromb Vasc Biol.

[24] Zhang Y, Gan W, Tian C, Li H, Lin X, Chen Y (2013). Association of PPP1R3B polymorphisms with blood lipid and C-reactive protein levels in a Chinese population (PPP1R3B C ). J Diabetes.

[25] Franceschini N, Carty C, Bůzková P, Reiner AP, Garrett T, Lin Y (2011). Association of genetic variants and incident coronary heart disease in multiethnic cohorts: the PAGE study. Circ Cardiovasc Genet.

[26] Adams HP, Bendixen BH, Kappelle LJ, Biller J, Love BB, Gordon DL (1993). Classification of subtype of acute ischemic stroke. Definitions for use in a multicenter clinical trial. TOAST. Trial of org 10172 in acute stroke treatment. Stroke.

[27] Wu DF, Yin RX, Cao XL, Huang F, Wu JZ, Chen WX (2016). MADD-FOLH1 polymorphisms and their haplotypes with serum lipid levels and the risk of coronary heart disease and ischemic stroke in a Chinese Han population. Nutrients.

[28] Cao XL, Yin RX, Huang F, Wu JZ, Chen WX (2016). Chromosome 9p21 and ABCA1 genetic variants and their interactions on coronary heart disease and ischemic stroke in a Chinese Han population. Int J Mol Sci.

[29] Chalmers J, MacMahon S, Mancia G, Whitworth J, Beilin L, Hansson L (1999). 1999 World Health Organization-International Society of Hypertension Guidelines for the management of hypertension. Guidelines sub-committee of the World Health Organization. Clin Exp Hypertens.

[30] Willer CJ, Schmidt EM, Sengupta S, Peloso GM, Gustafsson S, Kanoni S (2013). Discovery and refinement of loci associated with lipid levels. Nat Genet.

[31] Lettre G, Palmer CD, Young T, Ejebe KG, Allayee H, Benjamin EJ (2011). Genome-wide association study of coronary heart disease and its risk factors in 8,090 African Americans: the NHLBI CARe project. PLoS Genet.

[32] Coram MA, Duan Q, Hoffmann TJ, Thornton T, Knowles JW, Johnson NA (2013). Genome-wide characterization of shared and distinct genetic components that influence blood lipid levels in ethnically diverse human populations. Am J Hum Genet.

[33] Fedele F, Mancone M, Chilian WM, Severino P, Canali E, Logan S (2013). Role of genetic polymorphisms of ion channels in the pathophysiology of coronary microvascular dysfunction and ischemic heart disease. Basic Res Cardiol.

[34] Carty CL, Buzková P, Fornage M, Franceschini N, Cole S, Heiss G (2012). Associations between incident ischemic stroke events and stroke and cardiovascular disease-related genome-wide association studies single nucleotide polymorphisms in the population architecture using genomics and epidemiology study. Circ Cardiovasc Genet.

[35] Miao L, Yin RX, Huang F, Chen WX, Cao XL, Wu JZ (2017). The effect of MVK-MMAB variants, their haplotypes and GxE interactions on serum lipid levels and the risk of coronary heart disease and ischemic stroke. Oncotarget.

[36] Huang KK, Yin RX, Zeng XN, Huang P, Lin QZ, Wu J (2013). Association of the rs7395662 SNP in the MADD-FOLH1 and several environmental factors with serum lipid levels in the Mulao and Han populations. Int J Med Sci.

[37] Fedele F, Severino P, Bruno N, Stio R, Caira C, D'Ambrosi A (2013). Role of ion channels in coronary microcirculation: a review of the literature. Futur Cardiol.

[38] Yin RX, Zhou YJ, Hong SC (2014). Polymorphisms in the glucokinase regulator gene are associated with serum lipid levels and the risk of coronary artery disease and ischemic stroke. J Am Coll Cardiol.

[39] Yin RX, Yang Q, Zhou YJ (2014). Polymorphisms in the FADS1/FADS2 gene cluster are associated with the risk of coronary artery disease and ischemic stroke. J Am Coll Cardiol.

[40] Yang Q, Zhou YJ, Yin RX (2015). Polymorphisms in the MAFB gene are associated with the risk of coronary artery disease and ischemic stroke. J Am Coll Cardiol.

[41] Zhou YJ, Yin RX, Hong SC, Yang Q, Cao XL, Chen WX (2017). Association of the HNF1A polymorphisms and serum lipid traits, the risk of coronary artery disease and ischemic stroke. J Gene Med.

[42] Wu DF, Yin RX, Cao XL, Chen WX (2014). Association between single nucleotide polymorphism rs1044925 and the risk of coronary artery disease and ischemic stroke. Int J Mol Sci.

[43] Shi Y, He L (2005). SHEsis, a powerful software platform for analyses of linkage disequilibrium, haplotype construction, and genetic association at polymorphism loci. Cell Res.

[44] Dehghan A, Dupuis J, Barbalic M, Bis JC, Eiriksdottir G, Lu C (2011). Meta-analysis of genome-wide association studies in > 80 000 subjects identifies multiple loci for C-reactive protein levels. Circulation.

[45] Ligthart S, de Vries PS, Uitterlinden AG, Hofman A, CHARGE inflammation working group, Franco OH, Chasman DI, Dehghan A, et al. Pleiotropy among common genetic loci identified for cardiometabolic disorders and C-reactive protein. PLoS One. 2015, 10:e0118859. 10.1371/journal.pone.0118859 PMC4358943 25768928

[46] López-Mejías R, Genre F, Remuzgo-Martínez S, González-Juanatey C, Robustillo-Villarino M, Llorca J (2016). Influence of elevated-CRP level-related polymorphisms in non-rheumatic Caucasians on the risk of subclinical atherosclerosis and cardiovascular disease in rheumatoid arthritis. Sci Rep.

