# Supplementary material for: Association between the PPP1R3B polymorphisms and serum lipid traits, the risk of coronary artery disease and ischemic stroke in a southern Chinese Han population
Source: Nutr Metab (Lond). 2018 Apr 12;15:27. doi: 10.1186/s12986-018-0266-y (PMC5898016; doi:10.1186/s12986-018-0266-y)
Supplement: Supplementary file 1 — Table S1. Genotypic and allelic frequencies of the PPP1R3B polymorphisms in control and patients [n (%)]. Table S2. Association between the PPP1R3B genotypes and BMI or blood pressure in controls. Table S3. Linkage disequilibrium analysis of the 4 PPP1R3B SNPs. Table S4. Haplotype analysis of the 4 PPP1R3B SNPs. Figure S1. Annotation of SNPs in UCSC. Non-coding (ncRNA) and untranslated region (3’UTR) are colored blue. (DOC 392 kb) [file 12986_2018_266_MOESM1_ESM.doc]

**Supplementary Table 1** Genotypic and allelic frequencies of the *PPP1R3B* polymorphisms in control and patients [n (%)]

| SNP/Genotype/Allele | Control | CAD | IS | *P*_CAD_ | *P*_IS_ |
| --- | --- | --- | --- | --- | --- |
| rs12785 |  |  |  |  |  |
| TT | 356(58) | 286(51) | 280(53) |  |  |
| TA | 222(36) | 222(40) | 202(38) |  |  |
| AA | 39(6) | 48(9) | 49(9) | 0.067 | 0.093 |
| T | 934(76) | 794(71) | 762(72) |  |  |
| A | 300(24) | 318(29) | 300(28) | 0.019 | 0.032 |
| *P*_HWE_ | 0.58 | 0.60 | 0.16 |  |  |
| rs330910 |  |  |  |  |  |
| AA | 396(64) | 324(58) | 328(62) |  |  |
| AT | 194(31) | 200(36) | 170(32) |  |  |
| TT | 27(4) | 32(6) | 33(6) | 0.103 | 0.343 |
| A | 986(80) | 848(76) | 826(78) |  |  |
| T | 248(20) | 264(24) | 236(22) | 0.033 | 0.213 |
| *P*_HWE_ | 0.60 | 0.88 | 0.09 |  |  |
| rs330915 |  |  |  |  |  |
| AA | 409(66) | 345(62) | 329(62) |  |  |
| AT | 187(30) | 179(32) | 175(33) |  |  |
| TT | 21(3) | 32(6) | 27(5) | 0.094 | 0.183 |
| A | 1005(81) | 869(78) | 833(78) |  |  |
| T | 229(19) | 243(22) | 229(22) | 0.047 | 0.072 |
| *P*_HWE_ | 0.94 | 0.18 | 0.55 |  |  |
| rs9949 |  |  |  |  |  |
| GG | 357(58) | 286(51) | 279(53) |  |  |
| GA | 222(36) | 222(40) | 203(38) |  |  |
| AA | 38(6) | 48(9) | 49(9) | 0.054 | 0.067 |
| G | 936(76) | 794(71) | 761(72) |  |  |
| A | 298(24) | 318(29) | 301(28) | 0.014 | 0.023 |
| *P*_HWE_ | 0.66 | 0.60 | 0.18 |  |  |

SNP, single nucleotide polymorphisms; CAD, coronary artery disease; IS, ischemic disease; HWE, Hardy-Weinberg equilibrium; *P*_HWE_, the *P* value of the Hardy-Weinberg equilibrium; *P*_CAD_/*P*_IS_, the *P* value between patients and control.

**Supplementary Table 2** Association between the *PPP1R3B* genotypes and BMI or blood pressure in controls

| Genotype | n | BMI | SBP | DBP | PP |
| --- | --- | --- | --- | --- | --- |
|  |  | (kg/m^2^) | (mmHg) | (mmHg) | (mmHg) |
| rs12785 |  |  |  |  |  |
| TT | 356 | 23.61±3.12 | 132.87±22.26 | 80.55±12.43 | 52.32±16.31 |
| TA | 222 | 23.91±3.50 | 129.34±21.02 | 78.07±13.68 | 51.21±15.49 |
| AA | 39 | 23.64±3.35 | 132.20±21.97 | 79.39±12.52 | 52.80±16.68 |
| *P* |  | 0.566 | 0.163 | 0.083 | 0.658 |
| TT | 356 | 23.61±3.12 | 132.87±22.26 | 80.55±12.43 | 52.32±16.31 |
| TA+AA | 261 | 23.88±3.48 | 129.78±21.18 | 78.27±12.50 | 51.41±15.63 |
| *P* |  | 0.332 | 0.081 | 0.064 | 0.479 |
| rs330910 |  |  |  |  |  |
| AA | 396 | 23.65±3.12 | 132.00±21.95 | 80.11±12.54 | 51.90±15.90 |
| AT | 194 | 23.95±3.57 | 130.98±21.44 | 78.62±13.53 | 52.25±16.24 |
| TT | 27 | 23.11±3.25 | 129.19±23.36 | 78.56±14,25 | 50.63±16.71 |
| *P* |  | 0.343 | 0.735 | 0.390 | 0.881 |
| AA | 396 | 23.65±3.12 | 132.00±21.95 | 80.11±12.54 | 51.90±15.90 |
| AT+TT | 221 | 23.85±3.54 | 130.76±21.63 | 78.61±13.58 | 52.05±16.26 |
| *P* |  | 0.455 | 0.500 | 0.170 | 0.909 |
| rs330915 |  |  |  |  |  |
| AA | 409 | 23.68±3.16 | 132.34±22.08 | 80.15±12.82 | 52.13±16.43 |
| AT | 187 | 23.78±3.47 | 129.33±21.08 | 77.96±13.01 | 51.37±16.00 |
| TT | 21 | 24.03±3.86 | 136.24±22.71 | 82.62±13.50 | 53.62±16.43 |
| *P* |  | 0.856 | 0.179 | 0.086 | 0.769 |
| AA | 409 | 23.68±3.16 | 132.34±22.08 | 80.15±12.82 | 52.13±16.43 |
| AT+TT | 208 | 23.81±3.51 | 130.02±21.30 | 78.43±13.11 | 51.60±16.01 |
| *P* |  | 0.648 | 0.213 | 0.117 | 0.695 |
| rs9949 |  |  |  |  |  |
| GG | 357 | 23.61±3.13 | 132.88±22.25 | 80.54±12.44 | 52.34±16.29 |
| GA | 222 | 23.91±3.50 | 129.34±21.02 | 78.06±13.66 | 51.18±15.50 |
| AA | 38 | 23.65±3.34 | 132.18±21.95 | 79.36±12.50 | 52.82±16.66 |
| *P* |  | 0.565 | 0.163 | 0.081 | 0.656 |
| GG | 357 | 23.61±3.13 | 132.88±22.25 | 80.54±12.44 | 52.34±16.29 |
| GA+AA | 260 | 23.87±3.47 | 129.76±21.15 | 78.26±13.48 | 51.42±15.66 |
| *P* |  | 0.331 | 0.080 | 0.062 | 0.482 |

BMI, Body mass index; BP, Blood pressure; SBP, Systolic blood pressure; DBP, Diastolic blood pressure; PP, Pulse pressure. A *P* < 0.0025 was considered statistically significant after Bonferroni correction.

**Supplementary Table 3** Linkage disequilibrium analysis of the 4 *PPP1R3B* SNPs

| *D'* statistic |  |  |  | *r*^2^ statistic |  |  |  |
| --- | --- | --- | --- | --- | --- | --- | --- |
| SNP | rs330910 | rs330915 | rs9949 | SNP | rs330910 | rs330915 | rs9949 |
| rs12785 | 0.997 | 0.975 | 0.998 | rs12785 | 0.751 | 0.669 | 0.991 |
| rs330910 | - | 0.673 | 0.997 | rs330910 | - | 0.423 | 0.752 |
| rs330915 | - | - | 0.975 | rs330915 | - | - | 0.671 |
| rs9949 | - | - | - | rs9949 | - | - | - |

SNP, single nucleotide polymorphisms

**Supplementary Table 4** Haplotype analysis of the 4 *PPP1R3B* SNPs

| Haplotype | Control (freq) | CAD (freq) | OR(95%CI)_CAD_ | *P*_CAD_ | IS(freq) | OR(95%CI)_IS_ | *P*_IS_ |
| --- | --- | --- | --- | --- | --- | --- | --- |
| T-A-A-G | 931.77(0.753) | 790.81(0.711) | 0.80(0.66-0.96) | 0.014 | 755.64(0.712) | 0.81 (0.67-0.93) | 0.023 |
| A-T-T-A | 176.02(0.143) | 186.91(0.168) | 1.28(1.04-1.52) | 0.086 | 160.90(0.152) | 1.08 (0.85-1.36) | 0.541 |
| A-T-A-A | 70.94(0.057) | 76.04 (0.068) | 1.20(0.86-1.28) | 0.278 | 75.10(0.071) | 1.25 (0.89-1.75) | 0.193 |
| A-A-T-A | 49.80(0.040) | 53.95 (0.049) | 1.21(0.82-1.80) | 0.338 | 62.74(0.059) | 1.49 (1.03-2.19) | 0.036 |
| Rare | * |  |  |  |  |  |  |

**
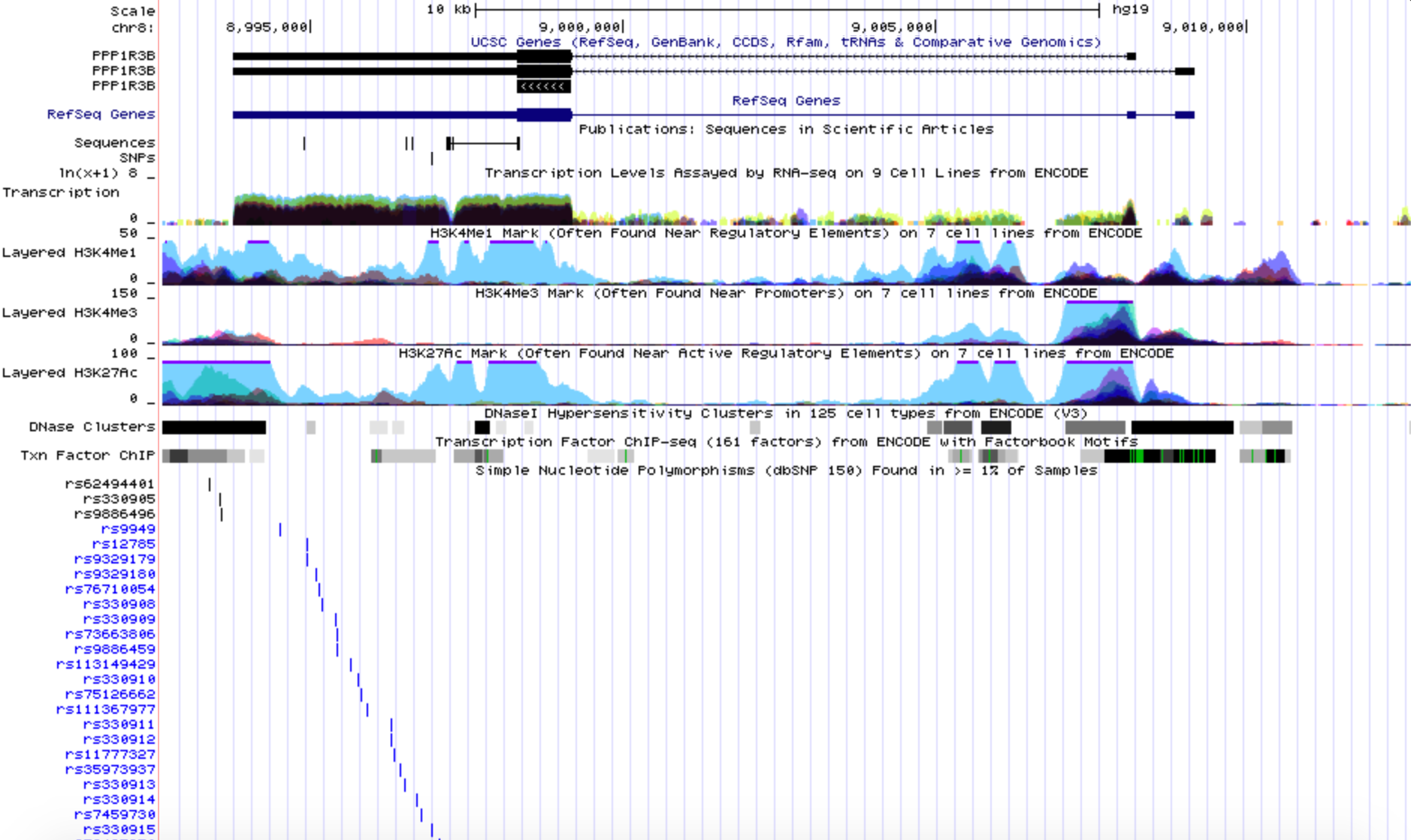
**

**Supplementary Figure 1** Annotation of SNPs in UCSC. Non-coding (ncRNA) and untranslated region(3’UTR) are colored blue.
